# Pharmacist care in hypertension management: systematic review of randomized controlled trials

**DOI:** 10.1093/eurpub/ckac131.132

**Published:** 2022-10-25

**Authors:** V Gastens, S Tancredi, B Kiszio, C Del Giovane, R Tsuyuki, G Paradis, A Chiolero, V Santschi

**Affiliations:** 1 Institute of Primary Health Care, University of Bern, Bern, Switzerland; 2 Population Health Laboratory, University of Fribourg, Fribourg, Switzerland; 3 La Source, School of Nursing Sciences, HES-SO University of Applied Sciences and Arts, Lausanne, Switzerland; 4 EPICORE Centre, University of Alberta, Edmonton, Canada; 5 School of Population and Global Health, McGill University, Montreal, Canada

## Abstract

**Background:**

Hypertension management remains a major public health challenge in primary care. Recent hypertension guidelines recommend the involvement of pharmacists for team-based care management of hypertension. Our objective is to systematically review the evidence of the impact of pharmacist care alone, or in collaboration, on BP amongst hypertensive outpatients compared with usual care. One major focus is to assess the heterogeneity in the effects of these interventions to identify which ones work best in a given healthcare setting.

**Methods:**

In collaboration with a medical librarian, a systematic literature search was conducted for any article published up to 22.10.2021 in MEDLINE, EMBASE, CENTRAL, CINAHL, Web of Science, and Trip databases. Randomized controlled trials assessing the effect of pharmacist interventions on BP among outpatients were included. The outcomes are the change in BP, BP at follow-up, or BP control. Results will be synthesized descriptively and, if appropriate, will be pooled across studies to perform meta-analysis. We published the study protocol in BMJ Open.

**Results:**

A total of 1768 study records were identified by electronic database searching and loaded to the systematic review management software Covidence. After removal of duplicates, 1744 were independently screened based on title and abstract by two authors (VG, ST), and 242 full texts were evaluated. A total of 72 studies with 32641 patients are currently included for data extraction. These studies were published between 1973 and 2021 and conducted in different regions (North America: n = 34, Europe: n = 13, other: n = 25). The data extraction and analysis are ongoing. Results will be presented at the congress.

**Conclusions:**

This systematic review provides updated evidence on the effect of pharmacist intervention on BP management. Heterogeneity in the effect of interventions will be carefully evaluated which will help the implementation of effective interventions in various healthcare settings.

**Key messages:**

• Recent hypertension guidelines recommend the involvement of pharmacists for team-based care management of hypertension.

• This systematic review provides updated evidence on the effect of pharmacist intervention on blood pressure management.

